# *Gynura procumbens*: An Overview of the Biological Activities

**DOI:** 10.3389/fphar.2016.00052

**Published:** 2016-03-15

**Authors:** Hui-Li Tan, Kok-Gan Chan, Priyia Pusparajah, Learn-Han Lee, Bey-Hing Goh

**Affiliations:** ^1^Biomedical Research Laboratory, Jeffrey Cheah School of Medicine and Health Sciences, Monash University MalaysiaBandar Sunway, Malaysia; ^2^Division of Genetic and Molecular Biology, Faculty of Science, Institute of Biological Sciences, University of MalayaKuala Lumpur, Malaysia

**Keywords:** *Gynura procumbens*, medicinal plant, biological activity, ethnopharmacology, Bai Bing Cao

## Abstract

*Gynura procumbens* (Lour.) Merr. (Family Asteraceae) is a medicinal plant commonly found in tropical Asia countries such as China, Thailand, Indonesia, Malaysia, and Vietnam. Traditionally, it is widely used in many different countries for the treatment of a wide variety of health ailments such as kidney discomfort, rheumatism, diabetes mellitus, constipation, and hypertension. Based on the traditional uses of *G. procumbens*, it seems to possess high therapeutic potential for treatment of various diseases making it a target for pharmacological studies aiming to validate and provide scientific evidence for the traditional claims of its efficacy. Although there has been considerable progress in the research on *G. procumbens*, to date there is no review paper gathering the reported biological activities of *G. procumbens*. Hence, this review aims to provide an overview of the biological activities of *G. procumbens* based on reported *in vitro* and *in vivo* studies. In brief, *G. procumbens* has been reported to exhibit antihypertensive, cardioprotective, antihyperglycemic, fertility enhancement, anticancer, antimicrobial, antioxidant, organ protective, and antiinflammatory activity. The commercial applications of *G. procumbens* have also been summarized in this paper based on existing patents. The data compiled illustrate that *G. procumbens* is a potential natural source of compounds with various pharmacological actions which can be utilized for the development of novel therapeutic agents.

## Introduction

*Gynura procumbens* (Lour.) Merr. (Family Asteraceae), is a small plant ~1–3 m in height. The stems are fleshy and the leaves are ovate-elliptic or lanceolate (Rahman and Asad, 2013). It has scientific synonym names such as *Gynura sarmentosa* DC and *Cacalia sarmentosa* Blume (Quattrocchi, 2012). The plant leaf is commonly consumed and scientifically it has been shown to be safe for consumption (Rosidah et al., 2008). In Malaysia, the fresh leaves of *G. procumbens* are commonly eaten raw and in Thailand, the leaves are also used for cooking (Kaewseejan et al., 2015). In Malay, *G. procumbens* is called Sambung Nyawa which means “prolongation of life” whereas in Chinese, it is called Bai Bing Cao which means “100 ailments” (Bodeker et al., 2009). This is because it has been utilized in traditional medicine both systemically and for topical application for treatment of different types of illnesses and diseases (Krishnan et al., 2015). For example, it is widely used to relieve kidney discomfort in Indonesia and people have been using it for the treatment of fever in Vietnam. In Thailand, it is commonly used to alleviate inflammation, rheumatism, and to cure viral ailments (Wiart, 2006). The beneficial properties of *G. procumben*s have been attributed to the presence of bioactive compounds such as flavonoids and glycosides in this plant (Akowuah et al., 2001, 2002).

Throughout the years, intensive research done on *G. procumbens* has provided extensive scientific evidence of its therapeutic potential. The present review aims to provide an overview of the biological activities of *G. procumbens* with reference to the available literature. The vast potential medical applications of *G. procumbens* based on the consolidation of the scientific findings of research on this plant are also highlighted.

## Biological activities

### Antihypertensive and cardioprotective activity

Hypertension is a key risk factor for several cardiovascular diseases including coronary vascular disease and stroke (Lu et al., 2012). Over the past few decades, significant effort has been expended to explore medicinal plants with antihypertensive therapeutic effect, including *G. procumbens* (Lam et al., 1998). To date, the administration of *G. procumbens* has been reported to result in significant lowering of systolic blood pressure and mean arterial pressure in hypertensive rats (Hoe and Lam, 2005; Kim et al., 2006; Hoe et al., 2007). Additionally, the treatment with *G. procumbens* extract has also resulted in significant decrease in heart rate, strong negative chronotropic, and negative ionotropic effects at rats' right atrium and left atrium respectively (Hoe et al., 2011; Kaur et al., 2012; Abrika et al., 2013).

Angiotensin (Ang)-converting enzyme is important for blood pressure regulation. It converts Ang I into Ang II, the peptide hormone with potent vasoconstrictive effects (Lote, 2006). Interestingly, the blood pressure-lowering effect of *G. procumbens* was associated with its inhibitory effect on angiotensin-converting enzyme activity (Hoe et al., 2007). In addition to that, *G. procumbens* has direct inhibitory effect on the activity of Ang II. This was demonstrated in the study that reported the inhibition of Ang II- induced contraction of aortic rings treated with fraction of *G. procumbens* (Poh et al., 2013). In terms of mechanism of action, *G. procumbens* possibly antagonizes the vasoconstrictive effect of Ang II through endothelium-dependent pathways that involve the activation of nitric oxide and prostaglandin release (Poh et al., 2013). This is supported by the reduction of inhibitory effect of *G. procumbens* aqueous fraction on Ang II-induced contraction in the presence of cyclooxygenase inhibitor and endothelial nitric oxide synthase inhibitor (Poh et al., 2013). This was also supported by another research finding which found increased serum nitric oxide level in hypertensive rats treated with *G. procumbens* extract (Kim et al., 2006).

Another possible mechanism of the vasodilatory effect of *G. procumbens* is inhibition of extracellular Ca^2+^ influx. Vasocontraction caused by phenylephrine, calcium and potassium chloride was shown to be antagonized by *G. procumbens* extracts. This was probably due to the blocking of receptor-operated and/or voltage dependent calcium channels as well as opening of potassium channel (Hoe et al., 2011; Ng et al., 2013). Therefore, *G. procumbens* potentially serves as an antihypertensive agent with cardioprotective activity due to its ability to target various mechanisms including the renin-angiotensin system and calcium influx which are crucial players in the pathophysiology of hypertensive conditions.

### Antihyperglycemic activity

*G. procumbens* is commonly used for diabetes treatment in traditional medicine and its hypoglycemic effect has been reported in *in vivo* studies (Hamid et al., 2004; Algariri et al., 2014). One intriguing finding on *G. procumbens*, is its specificity in inducing hypoglycemic effect only in diabetic animals as it has been shown to cause a significant decrease in fasting blood glucose levels and suppression of glucose elevation during glucose tolerance test in diabetic rats but not normal rats (Zhang and Tan, 2000; Algariri et al., 2013).

The effect of *G. procumbens* treatment on insulin level has been investigated. Hamid et al. (2004) has reported the stimulation of insulin secreting cell lines by *G. procumbens* extract. However, the exposure of clonal pancreatic cells with extract of *G. procumbens* did not stimulate insulin secretion (Hassan et al., 2008). These contradicting results might be due to the differing response of different cell lines when treated with *G. procumbens.* Therefore, its effect on insulin secretion has been further tested using *in vivo* studies. However, no significant change has been observed in plasma insulin level in diabetic rats treated with the extracts, implying that the hypoglycemic activity of *G. procumbens* does not rely on insulinotropic activity but may instead be due to its extra-pancreatic effect (Hassan et al., 2008; Lee et al., 2012).

Other pathways explored in the research include experiments on the antidiabetic effect of *G. procumbens* focusing on glucose uptake activity. The findings showed *G. procumbens* treatment stimulated glucose uptake on 3T3 adipocytes. Furthermore, an enhancement of activity was found in the presence of insulin (Bohari et al., 2006). Further validation was then conducted via *in vivo* work. The report showed an enhanced uptake of glucose by muscle tissue of diabetic rats, indicating a direct effect of *G. procumbens* extract on glucose uptake and utilization at the peripheral levels (Hassan et al., 2010).

With reference to metabolic pathways, *G. procumbens* was found to exert an effect on glucose metabolism in liver. It was demonstrated to cause phosphorylation and inactivation of glycogen synthase kinase 3 (GSK3) in the liver of diabetic rats, suggesting that the hypoglycemic action of *G. procumbens* is due to either direct or indirect effects on the upstream component(s) activities in the insulin signaling pathway (Gansau et al., 2012). In addition, it stimulated an increase in activity of glucokinase and pyruvate dehydrogenase and phosphorylation of ATP-citrate which are known to play roles in glucose metabolism (Kang et al., 2015). Furthermore, an enhancement of liver hexokinase, phosphofructokinase and fructose-1,6-bisphosphatase specific activity were also found following treatment. This indicates *G. procumbens* stimulated an increase in utilization of hepatic glucose and decreased endogenous glucose production (Lee et al., 2012).

There has also been work examining the hypoglycemic effect of *G. procumbens* in combination with other herbal therapies. It was observed to achieve a stronger hypoglycemic effect when *G. procumbens* was used together with *Azadirachta indica* or *Andrographis paniculata* (Pramono and Nugroho, 2015). The synergistic effect is postulated to be related to the diverse range of active compounds present in the extract combination (Sunarwidhi et al., 2014). Taken altogether, the current evidence suggests the presence of bioactive principles which possess insulin mimetic properties in *G. procumbens* (Hassan et al., 2010).

### Sexual and reproductive function enhancement activity

Aside from direct treatment of diabetes, research on *G. procumbens* has also explored its potential in treating infertility, which is one of the complications of diabetes (Ramalho-Santos et al., 2008). *G. procumbens* was found to exhibit an effect on sexual and reproductive function as the treatment with *G. procumbens* significantly increased sperm count, sperm motility, and reduced the percentage of sperm mortality of diabetic rats (Sani et al., 2008). *G. procumbens* was also demonstrated to have the aphrodisiac properties as evidenced by an increase in mounting frequency of diabetic rats following the treatment (Noor and Radzuan, 2012). In term of enzymatic activity, *G. procumbens* was found to promote testicular lactate dehydrogenase activity (Hakim et al., 2008). This finding can be correlated to improved fertility because lactate dehydrogenase is known to play a crucial role in spermatogenesis (Kaur and Bansal, 2004). Overall, studies clearly suggest that *G. procumbens* may improve the reproductive function of infertile diabetic males, particularly through an increase in sperm counts, quality, and motility.

### Anticancer activity

*G. procumbens* has long been used as traditional treatment for cancers such as leukemia, uterine, and breast cancers (Agustina et al., 2006). This has prompted scientific exploration of the antitumor activity of *G. procumbens* (Maw et al., 2011). Short term (10 weeks) treatment of the ethanolic extract was found to suppress the progression of nitroquinoline 1-oxide-induced tongue carcinogenesis during initiation phase. Longer period (26 weeks) of administration was demonstrated to lead to high suppression of oral carcinogenesis (Agustina et al., 2006). The ethanolic extract was also shown to be effective against carcinogenetic effect of 7,12-dimethylbenz(a)antracene on liver (Nisa et al., 2012). *G. procumbens* has been also tested on osteosarcoma cell line. The treatment has resulted in inhibition of cell proliferation and was observed to suppress the invasive and migratory abilities of the cancer cells (Wang et al., 2013). Recently, ethanolic extract of *G. procumbens* was shown to cause about 80% decrease in azoxymethane-induced aberrant crypt foci in rats which indicates potential in preventing colon cancer (Shwter et al., 2014). *G. procumbens* has also demonstrated its potential in preventing breast cancer. It was shown to cause effective suppression in proliferation of breast cancer and epithelial cells of mammary glands. Besides, the further studies conducted has proven that the treatment of *G. procumbens* was able to reduce the tumor incidence in the animals tested (Meiyanto et al., 2007; Hew et al., 2013; Gofur et al., 2015).

Mechanistically, *G. procumbens* inhibits the initiation phase of carcinogenesis. The treatment with ethanolic extract caused a significant reduction in expression and activity of cytochrome P-450 enzymes such as CYP3A4, CYP1A2, and CYP1A1 (Afandi et al., 2014; Ghofur et al., 2015). This inhibition may lead to a lower risk of cancer as it will result in a reduction in the conversion of the respective procarcinogens to cancer triggers (Afandi et al., 2014). In addition, *G. procumbens* treatment has also been shown to stimulate expression of glutathione-transferase which is involved in the detoxification of carcinogenic compounds. These activities help to prevent cancer formation at its initiation phase (Hamid et al., 2009; Ghofur et al., 2015).

Cancer patients frequently consume herbal medicine as complementary and alternative medicine while undergoing chemotherapy (Cheng et al., 2010). In view of this, co-treatment studies of *G. procumbens* and chemotherapy drugs have been carried out. The combination of *G. procumbens* extract with doxorubicin or 5-fluorouracil resulted in strong synergistic effect against breast and colon cancer cells (Meiyanto and Jenie, 2007; Nurulita et al., 2011, 2012). However, co-treatment of *G. procumbens* with cisplatin appeared to be antagonistic as this combination failed to further suppress cancer cell proliferation (Nurulita et al., 2011). This demonstrates that the concomitant use of *G. procumbens* with different chemotherapy drugs might result in variable treatment efficacy.

In general, the blockade of angiogenesis pathways will result in inhibition of growth, invasion, and metastasis of tumor cells (Hamid et al., 2011). *G. procumbens* was shown to exhibit antiangiogenic activity as the treatment caused inhibition in expression of vascular endothelial growth factor and prevented formation of new blood vessels on fertilized chicken eggs (Jenie et al., 2006; Hamid et al., 2013).

Based on the reported studies, *G. procumbens* appears to be an effective chemotherapeutic agent against a wide range of cancer cell types and it exerts its anticancer activities via the modulation of various points of carcinogenesis including cancer initiation, cell proliferation, metastasis, and angiogenesis.

### Antimicrobial activity

The increasing incidence of resistant strains of malaria, viruses and also bacteria to currently available drugs makes the search for alternative therapeutics from herbal plants a key area of interest (Tan et al., 2015). The antiplasmodial activity of *G. procumbens* was first reported by Vejanan et al. (2012). The research shows that *G. procumbens* extract exhibits chemo-suppression effects toward malarial parasite strains of *Plasmodium falciparum* 3D7 and *Plasmodium berghei* NK65; possibly via direct inhibition of GSK3 or indirect action on pi3K/Akt pathway. Besides, the ethanolic extract of aerial plant parts has been demonstrated to exhibit virucidal and antireplicative activity against herpes simplex virus HSV-1 and HSV-2. This was validated in a clinical trial on patients with recurrent herpes labialis where treatment with *G. procumbens* herbal gels reduced the number of patients infected with HSV (Jarikasem et al., 2013). Meanwhile, the antibacterial activities of *G. procumbens* have also been tested with the extract exhibiting antibacterial activity against gram-positive and gram-negative bacteria such as *Bacillus cereus, Pseudomonas aeruginosa, Vibrio parahaemolyticus*, and *Salmonella typhi* (Rahman and Asad, 2013; Zheng et al., 2014). The antifungal activity of *G. procumbens* against fungi such as *Candida albicans* and *Aspergillus niger* was also observed. The findings of these studies have provided supporting evidence that substantiate the traditional use of *G. procumbens* in the treatment of infections by pathogens such as herpes simplex virus and malaria parasites (Kaewseejan et al., 2012; Nasir et al., 2015).

### Antioxidant activity

The antioxidant activity of *G. procumbens* extracts was assessed via DPPH assay to measure its free radical scavenging ability (Akowuah et al., 2009; Afandi et al., 2014). In a comparative study, the ethanol extract of *G. procumbens* exhibited the highest percentage of DPPH inhibition (52.81%) among different types of plant extracts that were tested (Maw et al., 2011). Meanwhile, the reductive ability of *G. procumbens* extract has also been tested by using ferric reducing assay which has further proven the antioxidant capacity possessed by this plant (Kaewseejan et al., 2015).

Further examination of the antioxidant activity via a range of different assays including trolox equivalent, β-carotene—linoleic acid, and xanthine oxidase inhibitory assays have also been explored. Based on the reported data, *G. procumbens* was found to display substantial antioxidant activity (Rosidah et al., 2008). Since lipid peroxidation is a common result of oxidative stress, the antioxidative effect of *G. procumbens* was revealed when it inhibited lipid peroxidation with the median effective concentration of 2.75 mg/mL (Luerang et al., 2010; Kumar and Pandey, 2013). In addition, the administration of methanol extract prior to oxidative stress induction was able to reverse the elevation of plasma lipid peroxidation in tested animals (Akowuah et al., 2012). In order to differentiate the antioxidative capacity of different parts of *G. procumbens*, a recent study was conducted by Krishnan et al. (2015). The study revealed that the root extract showed the highest antioxidant activity when compared to the other parts of the plant. Based on the studies, *G. procumbens* appears to be a potent source of natural antioxidants probably due to its high phenolic content (Rosidah et al., 2008).

### Organ protective effect

The protective effect of *G. procumbens* against damage of body tissues and organs has also been evaluated. *G. procumbens* was found to exert a gastroprotective effect as the administration of ethanolic extract significantly lessened the areas of ethanol-induced gastric ulcer in rats; with a reduction of submucosal edema and infiltration of leucocytes was observed (Mahmood et al., 2010). This finding has intrigued the researchers to further explore the protective effect of *G. procumbens.* In a study on skin damage, the antiphotoaging property of *G. procumbens* has been discovered as it was found to cause a significant inhibition in the expression of matrix metalloproteinases induced by ultraviolet irradiation in human dermal fibroflasts (Kim et al., 2011). The results obtained in both studies demonstrated that its protective effects might be associated with the ROS scavenging activity of *G. procumbens* (Mahmood et al., 2010; Kim et al., 2011).

*G. procumbens* is also known to be effective in preventing progressive renal diseases. The aqueous extract of plant was found to cause inhibition of mesangial cell proliferation and DNA synthesis. The suppression of regulator proteins for cell proliferation was found to be responsible for this observed effect (Lee et al., 2007). In addition, *G. procumbens* was found to have a hepatoprotective effect as it was shown to attenuate the ethanol-induced lipid accumulation in mice livers by modulating lipid metabolism-related genes, particularly via MAPK/SREBP-1c-dependent and -independent pathways (Li et al., 2015).

Based on these findings, *G. procumbens* has significant potential as an organoprotective agent; mainly due to its antioxidative properties which exert a regulatory effect at the level of gene expression.

### Antiinflammatory activity

In Thai folk medicine, *G. procumbens* is commonly used to treat inflammation (Wiart, 2006). It was shown to prevent the increase in ear thickness of mice caused by croton oil-induced inflammation (Iskander et al., 2002). Besides, topical application of ethanol extract on the wounds of tested animals showed significant dermal healing signs, less scar width, and considerable faster healing rate when compared with control group treated with saline (Zahra et al., 2011). Furthermore, the histological analysis has also revealed there is a lesser amount of inflammatory cells at the granulation tissue of wound area and higher amount of collagen with angiogenesis.

Recently, the immunomodulatory activity of *G. procumbens* has been tested using mice splenic cells. The treatment of ethanolic leaf extracts of *G. procumbens* at 0.1 and 1.0 μg/mL caused higher proliferation of CD4^+^CD25^+^, CD4^+^CD62L^−^, CD4^+^CD62L^+^, CD8^+^CD62L^−^, and CD8^+^CD62L^+^ T cells but lower proliferation of B220^+^ cells when compared to the higher dosage at 10 μg/mL. However, at dosage of 10 μg/mL, it was shown to promote high proliferation of B cells. These results have demonstrated that the concentration used in experiment is the determining factor for whether *G. procumbens* acts as an immunostimulant or immunosuppressant (Dwijayanti and Rifa'i, 2014, 2015).

Inflammation and the immune system are closely linked. For instance, the immune system plays a crucial role in the pathogenesis of the inflammatory disorder known as atherosclerosis which can be treated using statins—drugs with anti-inflammatory properties and immunomodulatory properties (Shovman et al., 2002). Therefore, the antiinflammatory and immunomodulatory activity of *G. procumbens* may be utilized for the treatment of inflammatory diseases or conditions that involve the immune system.

### Commercial uses

Among the existing patents related to *G. procumbens*, the majority of them are for preparations of traditional Chinese medicine intended for the treatment of various ailments including uterine cancer (Liao, 2015), cervical spondylosis (Shi, 2015), and chronic skin ulcer (Yang et al., 2015). Besides, it has also been used as an ingredient in special diets for patients with medical conditions such as heart (Chen et al., 2013b) and liver disease (Chen et al., 2013a). In the food industry, it has been incorporated into products such as tea (Hu, 2014; Liao et al., 2014; Liu, 2015), kimchi (Jang, 2013), coffee powder (Park, 2015), chocolate (Jang, 2014), candy (Xie, 2007c), and chewing gum (Xie, 2010). The applications of *G. procumbens* in personal care and cosmetic products have also been reported which including hand-washing solution (Xie, 2009), hand sanitizer (Xie, 2007a), oral spray (Xie, 2007b), facial masks (Yuan and She, 2014), and skin care creams (Xie, 2007d). These patents have demonstrated the high commercial value of *G. procumbens* and its variety of uses in a number of industries.

## Conclusion

**Figure 1:**
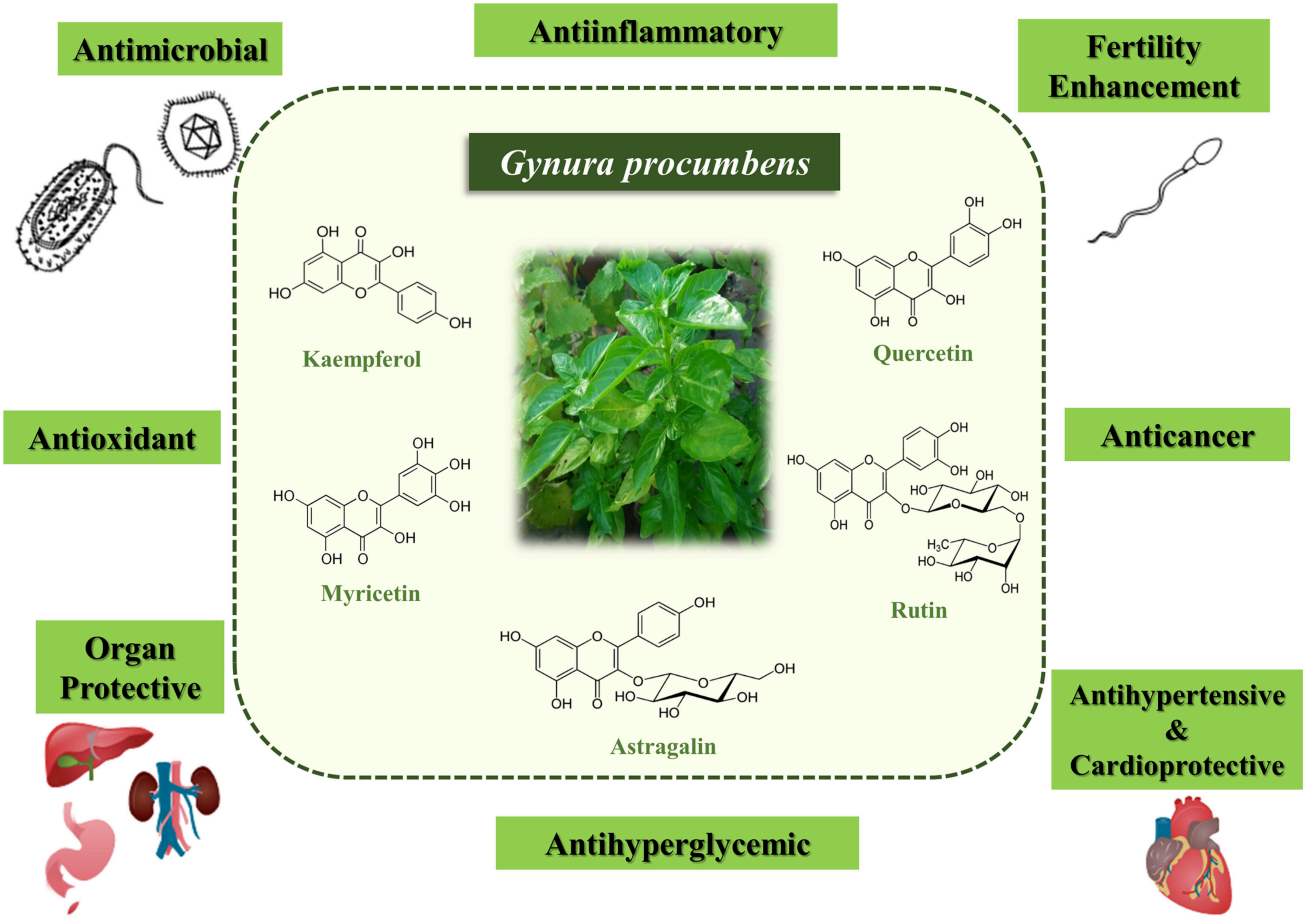
**Biological activities of *Gynura procumbens* and its main bioactive constituents that contributed to the biological activities**.

In summary, *G. procumbens* has been demonstrated to have high therapeutic value and has enormous potential for application in the development of medical treatments as well as consumer goods. Its diverse pharmacological effects and biological properties (Table 1) are mainly attributed to its flavonoid content (Figure 1). However, there is still limited knowledge regarding the underlying mechanisms of action and exact chemical constituents involved. Further research elucidating the mechanisms underlying the biological activities is needed for development of standardized drugs or herbal products.

**Table 1:** **Summary of biological activities of *Gynura procumbens***.

**Biological activities**	**Plant part**	**Type of extract**	**Tested dose**	Effective dose^*^	**Positive control**	**Description of activity**	**Possible mechanism of action**	**Class of compounds**	**Compound(s)**	**References**
Anticancer	Leaf	Ethanol	300, 750 mg/kg bw	300, 750 mg/kg bw	DMBA (20 mg/kg bw)	Reduced CYP1A1 expression and increased GSTμ expression.	Flavonoids might act as antagonist of Aryl hydrocarbon Receptor and caused inhibition of CYP1A1. Steroids might induce expression of GST through activation of the transcription factor glucocorticoid response element.	Flavonoids, Steroids	–	Hamid et al., 2009
	Leaf	Ethanol	300, 750 mg/kg bw	300, 750 mg/kg bw	DMBA (20 mg/kg bw)	Antiproliferative effect on liver cells of rats induced by DMBA.	Suppression on activity of cytochrome P-450 and induction of activity of GST.	–	–	Nisa et al., 2012
	Leaf	Ethanol	300, 750 mg/kg bw	–	DMBA	Decreased proliferation of mammary gland epithelial cells.	–	–	–	Hamid, 2009
	Leaf	Ethanol	300, 750 mg/kg bw	300, 750 mg/kg bw	DMBA (20 mg/kg bw)	Reduced CYP1A1 expression and increased GSTμ expression.	–	Flavonoids	–	Ghofur et al., 2015
	Leaf	Ethanol	25, 50, 100, 250, 500 μg/mL	–	–	Inhibition of breast cancer cells proliferation and potentiated efficacy of doxorubicin.	Inhibition of activities of P-glycoprotein and ATPase.	Flavonoids	–	Meiyanto and Jenie, 2007
	Leaf	Ethanol	250, 500, 750 mg/kg bw	250, 500, 750 mg/kg bw	DMBA (20 mg/kg bw)	Suppressed DMBA-induced breast cancer development in rats.	Suppression on activity of cytochrome P-450 and induction of activity of GST.	Flavonoids	–	Meiyanto et al., 2007
	Leaf	Ethanol	300, 750 mg/kg bw	–	DMBA (20 mg/kg bw/day)	Suppressed tumor incidence in DMBA treated rats.	–	–	–	Gofur et al., 2015
	Leaf	Ethanol	100, 1000 ppm	–	70% Ethanol	Absence of tumor growth (carrot-disc assay).	–	–		Maw et al., 2011
	Leaf	Ethanol	3.5 g dry leaves/kg bw	–	–	Inhibition of progression of 4NQO-induced rat tongue carcinogenesis during initiation phase.	Antioxidant and scavenging effect on activated carcinogens as well as action on protein that regulate the progression of cell cycle.	Flavonoids	–	Agustina et al., 2006
	Leaf	Ethanol	250, 500 mg/kg bw	250, 500 mg/kg bw (*p* < 0.001)	5-FU (35 mg/kg bw)	Reduced total azoxymethane-induced aberrant crypt foci in rats.	Detoxification by glutathione-S-transferase and reduction in oxidative stress or antiproliferative effect.	Phenolics	–	Shwter et al., 2014
	Leaf	Ethanol	10, 20, 40, 80 μg	10, 20, 40, 80 μg	Basic fibroblast growth factor (60 ng)	Inhibition of angiogenesis on chick CAM embryo.	Inhibition of COX-2 activity, prostaglandin synthesis, and MMP activity.	Flavonoids	–	Jenie et al., 2006
	Leaf	Ethanol	60, 75, 90, 110 μg	60, 75, 90, 110 μg	Basic fibroblast growth factor (60 ng)	Inhibition of angiogenesis on chick CAM embryo.	Inhibition of COX-1 activity, tyrosine kinase, and MMP activity.	Flavonoids	–	Hamid et al., 2011
	Leaf	Ethanol	60, 75, 90, 110 μg	75, 90, 110 μg	Basic fibroblast growth factor (60 ng)	Inhibition of VEGF expression on chick CAM embryo.	Inhibition of VEGF receptor through inhibition of COX-2, tyrosine kinase, and MMP activity.	Flavonoids	–	Hamid et al., 2013
	Leaf and stem	Ethanol	5, 10, 20, 40, 80, 160 μg/mL	–	–	Inhibition of osteosarcoma cell line proliferation and metastasis and apoptosis induction.	Inhibition of nuclear translocation of NF-kB.	–	–	Wang et al., 2013
	Leaf	Ethanol (Ethyl acetate fraction)	0–500 μg/mL	–	–	Inhibition on proliferation of breast cancer cells and potentiated efficacy of 5-FU and doxorubicin.	Modulation of microtubule integrity that led to cell cycle arrest and inhibition of cell proliferation.	–	–	Nurulita et al., 2012
	Leaf	Ethanol (Ethyl acetate fraction)	25, 50, 100, 250, 500 μg/mL	–	–	Inhibition of WiDr colon cancer cells proliferation and potentiated efficacy of 5-FU but antagonism effect with cisplastin.	Cell cycle modulation such as G1 and S phase arrests as well as apoptosis induction.	Flavonoids	β-sitosterol, Stigmasterol, Kaempferol-3-O-Rutinoside, Astragalin, Quercetin	Nurulita et al., 2011
	Leaf	Protein extract	5, 10, 15, 20, 25 μg/mL	–	–	Inhibition of breast cancer cells proliferation.	Down regulated expression of proliferation markers such as Ki67 and PCNA, as well as invasion markers, CCL2.	Proteins	Cu,Zn-SOD, TIR-NBS-LRR, Ascorbate peroxidase, Malate dehydrogenase	Hew et al., 2013
Antihyperglycemic	Leaf	Aqueous	1 g/kg bw	1 g/kg bw	Metformin (500 mg/kg bw)	Reduced fasting blood glucose levels in diabetic rats.	Extra-pancreatic action of *G. procumbens* extract.	Flavonoid and glycosides	Rutin, Quercetin, Kaempferol, Astragalin	Hassan et al., 2008
	Leaf	Aqueous	0.5, 1 g/kg bw	1 g/kg bw	Metformin (500 mg/kg bw)	Reduced fasting blood glucose levels in diabetic rats, increased muscle tissue glucose uptake.	*G. procumbens* extract that mimiced or improved the action of insulin at the cellular level.	Flavonoid and glycosides	Rutin, Quercetin, Kaempferol, Kaempferol-3-O-rutinoside, Astragalin	Hassan et al., 2010
	Leaf	Ethanol and Aqueous	50, 100, 150 mg/kg bw	50, 100, 150 mg/kg bw	Glibenclamide (5 mg/kg bw) and Metformin (500 mg/kg bw)	Reduced fasting blood glucose and HbA1c levels in diabetic rats, increased activities of liver hexokinase, phosphofructokinase and fructose-1,6-bisphosphatase.	Glucose metabolism through glycolytic pathway and inhibition of hepatic endogenous glucose production through the gluconeogenic pathway.	Flavonoid and glycosides	–	Lee et al., 2012
	Leaf	Ethanol	50, 150, 300 mg/kg bw	50, 150, 300 mg/kg bw	Glibenclamide (5 mg/kg bw) and Metformin (500 mg/kg bw)	Decreased serum glucose levels in diabetic rats.	Biguanide-like activity of *G. procumbens.*	–	–	Zhang and Tan, 2000
	Leaf	Ethanol	37.5, 75, 112.5 mg/kg bw	37.5, 75, 112.5 mg/kg bw	Glibenclamide (4.5 mg/kg bw)	Decreased blood glucose level, improved pancreatic islet condition, increased insulin expression.	Synergistic effect with *Andrographis paniculata* in lowering blood glucose. Antioxidants improved pancreatic β-cell distribution and blocked the nitric oxide synthase activity in pancreatic β-cell.	Phenolic and flavonoid compounds	Kaempferol, Quercetin, Astragalin	Pramono and Nugroho, 2015
	Leaf	Ethanol	37.5, 75, 112.5, 150 mg/kg bw	–	Glibenclamide (0.45 mg/kg bw)	Decreased blood glucose level, improved pancreatic islet condition, increased insulin expression.	Synergistic effect with *Azadirachta indica* in lowering blood glucose. Antioxidants protected β-cell pancreas from oxidative damage.	Flavonoids	Quercetin	Sunarwidhi et al., 2014
	Leaf	Ethanol	1 g/kg bw	1 g/kg bw	Metformin (500 mg/kg bw)	Reduced fasting blood glucose levels in diabetic rats.	Metformin-like mechanisms such as increased hepatic gluconeogenesis rates and enhanced insulin sensitivity.	Phenolic and flavonoid compounds	Chlorogenic acid	Algariri et al., 2013
	Leaf	Ethanol (Hexane, ethyl acetate and n-butanol)	250 mg/kg bw	250 mg/kg bw	Glibenclamide (5 mg/kg bw)	Reduced fasting blood glucose levels and inactivation of GSK-3β in liver of diabetic rats.	Direct or indirect actions on activities of upstream components of insulin biosignaling pathway.	Flavonoids and glycosides	Kaempferol, Kaempferol-3, 7-di-O-β-D-glucoside	Gansau et al., 2012
	Leaf	Ethanol (Ethyl acetate, n-butanol, aqueous)	500, 1000, 2000 mg/kg bw	500, 1000, 2000 mg/kg bw	Metformin (500 mg/kg bw)	Reduced fasting blood glucose levels in diabetic rats.	–	Phenolic and flavonoid compounds	–	Algariri et al., 2014
	Leaf	Methanol (Hexane, ethyl acetate and butanol fraction)	0.005, 0.01, 0.05, 0.1, 0.5 mg/mL	–	Insulin	Increased glucose uptake in 3T3-F442A adipocytes.	Stimulation of glucose uptake and insulin action potentiation.	–	–	Bohari et al., 2006
	Leaf	Methanol (Butanol fraction)	1 g/kg bw	1 g/kg bw	Glibenclamide (0.025 mg/kg bw)	Reduced fasting blood glucose levels in diabetic rats.	–	Flavonoids		Akowuah et al., 2002
	Leaf	Methanol	1 g/kg bw	–	–	Hypoglycemic effect in normal rats and stimulated insulin secretion in insulin secreting cells.	–	–	–	Hamid et al., 2004
	–	Aqueous	3 mg/mL	–	Acarbose	Increased activity of GK and PDH as well as increased expression of pACL and pGSK-3β. High α-glucosidase inhibition activity.	GK and PDH activation, induction of expression of pACL, pGSK-3β associated to glucose metabolism.	–		Kang et al., 2015
Antihypertensive and cardioprotective	Leaf	Aqueous	500 mg/kg bw	500 mg/kg bw	–	Reduced systolic blood pressure in hypertensive rats. Reduced serum lactate dehydrogenase, creatine phosphate kinase, and increased serum nitric oxide concentration.	Increased production of nitric oxide in blood vessel and caused vasodilation.	–	–	Kim et al., 2006
	Leaf	Aqeuous and Ethanol	0.25, 0.5, 1.0, 2.0 mg/mL	Vasorelaxation and Ionotropic: 1.0, 2.0 mg/mL; Chronotropic: 0.25, 0.5, 1.0 mg/mL	–	*In vitro* vasorelaxation of isolated aorta, negative chronotropic effect in right atrium and negative ionotropic effects in left atrium.	–	Flavonoids	–	Kaur et al., 2012
	Leaf	Ethanol (Aqueous fraction)	0–20 mg/kg bw	0–20 mg/kg bw	–	Decreased mean arterial pressure of hypertensive and normotensive rats as well as inhibition of ACE activity.	Ganglionic and muscarinic cholinergic receptors activation as well as inhibition of ACE activity.	–		Hoe and Lam, 2005
	Leaf	Ethanol (Aqueous fraction)	0.625, 1.25, 2.5, 5, 10 mg/kg bw	0.625, 1.25, 2.5, 5, 10 mg/kg bw	Captopril (20 μg/kg)	Decreased mean arterial pressure of hypertensive and normotensive rats. Inhibition of Ang I-induced mean arterial pressure rise and decreased ACE activity *in vitro*.	Inhibition of ACE activity and antagonistic actions on receptors of Ang II.	Glycoconjugates and peptides	–	Hoe et al., 2007
	Leaf	Ethanol (Aqueous fraction)	10 mg/kg bw	10 mg/kg bw	–	Decreased contraction of rat aortic rings evoked by Ang I and Ang II. Potentiation of vasorelaxant effect and blood pressure lowering effect of bradykinin *in vivo.*	Endothelium-dependent pathway that involves nitric oxide and prostaglandins release.	–	–	Poh et al., 2013
	Leaf	Ethanol (Butanol fraction)	2.5, 5, 10, 20 mg/kg bw	MAP: 2.5, 5, 10, 20 mg/kg bw; HR: 10, 20 mg/kg bw	–	Immediate decrease of mean arterial pressure and heart rate in rats.	Vasodilatation caused by inhibition of calcium influx through receptor-operated and/or voltage dependent calcium channels.	–	–	Hoe et al., 2011
	Leaf	Ethanol (Butanol fraction and sub-fractions)	10^−7^–10^−2^ mg/mL	10^−7^–10^−2^ mg/mL	–	Inhibition of rat aortic rings contractions induced by phenylephrine and potassium chloride. Antagonized calcium-induced vasocontractions.	Blocking of calcium channels, opening of potassium channels, and stimulation of prostacyclin release.	Flavonoids		Ng et al., 2013
	Leaf	Methanol (Butanol fractions and subfractions)	0.25, 0.5, 1.0 mg/mL	Methanol: 0.5 mg/mL, 1.0 mg/mL; Butanol Fraction: 0.5 mg/mL, 1.0 mg/mL; Butanol subfraction: 0.25, 0.5, 1.0 mg/mL	–	Anticontraction activity on the left atrium by promoting relaxation.	Direct effect on sinoatrial node that caused decrease in conduction or to the depression of heart myocardium.	–		Abrika et al., 2013
Antiinflammatory	Aerial	Ethyl acetate	0.75 mg/ear	0.75 mg/ear	Hydrocortisone (1–6 mg/ear)	Inhibition of ear inflammation.	–	–	–	Iskander et al., 2002
		Hexane and toluene fractions of ethyl acetate extract	0.75 mg/ear	0.75 mg/ear toluene; 0.75 mg/ear hexane (*p* < 0.001)		Inhibition of ear inflammation.	–	Essential oils, titerpenes/steroid, bitter principles	–	
	Leaf	Ethanol	100, 200 mg/mL	100, 200 mg/mL	Intrasite gel (0.2 mL)	Accelerated wound healing rate, less scar width, less inflammatory cells at granulation tissue, more collagen with angiogenesis.	Antimicrobial, antioxidant, antiinflammatory activity.	Flavonoids	–	Zahra et al., 2011
	Leaf	Ethanol	0.1, 1, 10 μg/mL	0.1, 1, 10 μg/mL	–	Increased proliferation of T cells.	Increased in release of cytokine such as IL-2 and IFNγ.	Flavonoids and saponin	–	Dwijayanti and Rifa'i, 2014
	Leaf	Ethanol	0.1, 1 μg/mL	0.1, 1 μg/mL	–	Increased proliferation of T cells and decreased proliferation of B cells.	Complex synergistic and antagonistic effect of flavonoids which affect the immunostimulator and immunosuppressant properties.	Flavonoids	–	Dwijayanti and Rifa'i, 2015
			10 μg/mL	10 μg/mL	–	Increased proliferation of B cells.				
Antimicrobial	Aerial	Ethanol	1%, 2% (Herbal Gels)	–	–	Virucidal action against HSV-1 and HSV-2, reduced infection of HSV-1 in clinical trial patients with recurrent herpes labialis.	Antiinflammatory effect that relieves the infectious symptoms.	Caffeoylquinic derivatives, glycoglycerolipids and phytosteryl glucosides	–	Jarikasem et al., 2013
	Leaf	Dichloromethane, Ethyl acetate	400 μg/disc	–	Kanamycin (30 μg/disc)	Antibacterial activity against Gram positive and Gram negative bacteria, antifungal activity.	–	–	–	Rahman and Asad, 2013
	Leaf	Ethanol, Aqueous	25, 50, 100, 250 mg/kg bw	25, 50, 100, 250 mg/kg bw	Chloroquine (10 mg/kg bw)	Suppressing growth of malarial parasites and increase survival time of infected mice.	Direct inhibitory action of GSK or indirect activation of PI3K/Akt pathway.	Flavonoids	Kaempferol, Quercetin	Vejanan et al., 2012
Antioxidant	Leaf	Methanol	1 g/kg bw	1 g/kg bw	–	Reversed plasma lipid peroxidation of rats produced by carbon tetrachloride.	Enhanced and maintained activity of antioxidant enzymes that combat free radicals.	Polyphenols	–	Akowuah et al., 2012
Organ protective	Leaf	Aqueous	50, 100 μg/mL	50, 100 μg/mL	Captopril (250 μM)	Inhibition of mesangial cell proliferation.	Suppression on expression of platelet-derived growth factor, transforming growth factor-β1, cyclin-dependent kinase 1 and cyclin-dependent kinase2.	–	–	Lee et al., 2007
	Leaf	Ethanol	1, 10, 20 μg/mL	1, 10, 20 μg/mL	Retinoic acid (10 μM)	Inhibition of UV-induced expression of MMP-1, MMP-9, IL-6, and IL-8.	Inhibition of ROS and pro-inflammatory cytokine overproduction.	Flavonol glycosides	Kaempferol, Quercetin derivaties	Kim et al., 2011
	Leaf	Ethanol	50, 100, 200, 400 mg/kg bw	50, 100, 200, 400 mg/kg bw	Omeprazole (20 mg/kg bw)	Reduction of ulcer areas in the gastric wall, reduction/absent of edema, and infiltration of leucocytes.	Antioxidant activity that involves scavenging of ROS and free radicals; enhancement of mucosal defense system.	Flavonoids	–	Mahmood et al., 2010
	Stem	Ethanol	12.5, 25, 50 mg/kg bw	50 mg/kg bw	–	Attenuated acute ethanol-induced serum alanine aminotransferase levels and hepatic lipid accumulation.	Modulation of lipid metabolism-related genes via MAPK/SREBP-1c-dependent and independent pathways.	Phenolic compounds	Chlorogenic acid	Li et al., 2015
		N-butyl alcohol fraction (60% ethanol eluted fraction)	10 mg/kg bw	10 mg/kg bw	Silymarin (100 mg/kg bw/day)	Attenuated chronic ethanol-induced serum alanine aminotransferase levels and hepatic lipid accumulation.				
Sexual and reproductive function enhancement	Leaf	Aqueous	100 mg/kg bw	100 mg/kg bw	Glibenclamide (5 mg/kg bw)	Increased sperm count, improved sperm mobility, reduced sperm mortality and increased testicular LDH.	Neutralized reaction oxygen species activity and inhibition of lipid peroxidation by blocking the activity of peroxyl radical.	Flavonoids	–	Hakim et al., 2008
	Leaf	Aqueous	50 mg/kg bw	–	–	Increased sperm count, improved sperm mobility and reduced sperm mortality.	–	–	–	Sani et al., 2008
	Leaf	Methanol (Ethyl acetate fraction)	50, 300 mg/kg bw	50, 300 mg/kg bw	Metformin (300 mg/kg bw)	Increased sperm count, improved sperm mobility and increased mounting frequency.	As a consequence of anti-hyperglycemia effect of *G. procumbens* extract.	Flavonoids	–	Noor and Radzuan, 2012

* *Effective Dose: Dose that gives significant results with p < 0.05, p < 0.01, or p < 0.001*.

*5-FU, 5-fluorouracil; ACE, angiotensin-converting enzyme; ACL, ATP-citrate lyase; Ang, angiotensin; CAM, chorioallantoic membrane; COX, cyclooxygenase; Cu,Zn-SOD, superoxide dismutase cooper zinc; DMBA, 7,12-dimethylbenz(a)antracene; GK, glucokinase; GSK, glycogen synthase kinase; GSTμ, gluthathione s-transferase μ; Hba1c, hemoglobinA1c; HR, heart rate; HSV, herpes simplex virus; MAP, mean arterial pressure; MMP, matrix metalloproteinase; PDH, pyruvate dehydrogenase; ROS, reactive oxygen species; TIR-NBS-LRR, Toll Interleukin 1 Receptor-Nucleotide Binding Site-Leu-Rich Repeat; VEGF, vascular endothelial growth factor*.

## Author contributions

All authors listed, have made substantial, direct and intellectual contribution to the work, and approved it for publication.

### Conflict of interest statement

The authors declare that the research was conducted in the absence of any commercial or financial relationships that could be construed as a potential conflict of interest.
